# Supplementary material for: Ensuring fair, safe, and interpretable artificial intelligence-based prediction tools in a real-world oncological setting
Source: Commun Med (Lond). 2023 Jun 22;3:88. doi: 10.1038/s43856-023-00317-6 (PMC10287624; doi:10.1038/s43856-023-00317-6)
Supplement: Supplementary file 2 — Supplementary Information [file 43856_2023_317_MOESM2_ESM.pdf]

Supplementary Information

Ensuring fair, safe, and interpretable artificial intelligence-based prediction tools in a real-world oncological setting

Renee George[1,†], Benjamin Ellis[1,†], Andrew West[1], Alex Graff[1], Stephen Weaver[1], Michelle Abramowski[1], Katelin Brown[1], Lauren Kerr[1], Sheng-Chieh Lu[2], Christine Swisher[1,3, ‡], Chris Sidey-Gibbons[2, ‡,*]

[1] The Ronin Project, San Mateo, CA, United States

[2] Section of Patient-Centered Analytic, Divison of Internal Medicine, The University of Texas MD Anderson Cancer Center, Houston, Texas, U.S.A.

[3] The Lawrence J. Ellison Institute for Transformative Medicine, Los Angeles, CA, U.S.A.

[†] These authors contributed equally

[‡] These authors jointly supervised this work

[*] Corresponding Author: cgibbons@mdanderson.org

1. Supplementary Methods
    a. Feature transformation
    b. The variational autoencoder - *k*NN algorithm (VAE-*k*NN)
2. Supplementary Table 1. Performance of evaluated models on the testing dataset.

Supplementary Methods

Feature transformation

One-hot encoding is a method for transforming categorical data into numerical data. Each distinct value in the column is transformed into a column in the output data set. A value of "1" in the array indicates the presence of the feature while a "0" indicates the absence. It is possible for a row to have many one-hot encoded variables (e.g. many patients have multiple comorbidities). Min-max scaling is a transformation of continuous variables such that their range is bounded [0, 1]. Bucketization is the process of generating categorical features from continuous ones. For example, all of the lab features in the data set are bucketized. Based on the raw value, it was determined if the measurement was "low", "normal", or "high". Then, as with one-hot encoding, a "1" indicated the measurement's interpretation while the rest were set to "0".

The variational autoencoder - *k*NN algorithm (VAE-*k*NN)

The VAE-*k*NN is a combination of two different algorithms. The variational autoencoder is a vital step of the algorithm because it addresses one of the drawbacks of *k*NN algorithms - sparse feature vectors. An autoencoder learns a denser, more compact representation of the input features. It does this by learning two functions, an encoder function and a decoder function. The autoencoder is trained by chaining the encoder-decoder functions such that the input data is satisfactorily reproduced. For the *k*NN portion, just the encoder can be used to reduce the feature space. This aids the nearest neighbors search because the curse of dimensionality is mitigated. The encoded feature representation is tuned using contrastive learning. Contrastive learning is a method by which the distance in encoded space between same-labeled data points is minimized while the distance in encoded space between differently-labeled data points is maximized. In principle, this should generate denser and purer groupings of samples based on their label in the latent space.

The *k*NN algorithm is a well understood algorithm and a natural choice for classification tasks where model interpretability is an important factor. The algorithm works by saving training features and their corresponding class labels. To generate a prediction for an unseen feature vector, we search for the *k* nearest data points (*k* being an optimizable parameter) based on some distance function to that query vector. The predicted label is then the majority vote based on the labels in the neighborhood of *k* training points. The *k*NN implementation here slightly differs from a standard implementation in that we constrain neighbor selection by only allowing a single row-per-

patient-identifier in the neighbor data points. This avoids the model overfitting to a single patient that may have many similar characteristics to a query data point.

Supplementary Table 1. Performance of evaluated models on the testing dataset.

Algorithm	AUC	Brier Score
Gradient-boosted machines	0.80 [0.79-0.81]	0.07 [0.07, 0.07]
Logistic Regression	0.79 [0.78-0.79]	0.07 [0.07, 0.07]
Random Forest	0.77 [0.77-0.78]	0.08 [0.08, 0.08]
Multinomial Naive Bayes	0.77 [0.76-0.78]	0.13 [0.12, 0.13]
k-Nearest Neighbors	0.76 [0.75-0.77]	0.07 [0.07, 0.08]
VAE *k*-Nearest Neighbor	0.80 [0.79-0.80]	0.07 [0.07, 0.07]

Abbreviations: AUC: Area under the receiver operating characteristics curve; and VAE : Variational Autoencoder
