# Supplementary material for: Ensuring fair, safe, and interpretable artificial intelligence-based prediction tools in a real-world oncological setting
Source: Commun Med (Lond). 2023 Jun 22;3:88. doi: 10.1038/s43856-023-00317-6 (PMC10287624; doi:10.1038/s43856-023-00317-6)
Supplement: Supplementary file 7 — Description of Additional Supplementary Files [file 43856_2023_317_MOESM7_ESM.pdf]

Description of Additional Supplementary Files

File Name: Supplementary Data 1

Description: Included features.

File Name: Supplementary Data 2

Description: Description of modeled features.

File Name: Supplementary Data 3

Description: Bucketization thresholds

File Name: Supplementary Data 4

Description: additional source data for figures 2, 3, & 4
