# Supplementary material for: Ensuring fair, safe, and interpretable artificial intelligence-based prediction tools in a real-world oncological setting
Source: Commun Med (Lond). 2023 Jun 22;3:88. doi: 10.1038/s43856-023-00317-6 (PMC10287624; doi:10.1038/s43856-023-00317-6)
Supplement: Supplementary file 8 — Reporting Summary [file 43856_2023_317_MOESM8_ESM.pdf]

nature portfolio

Corresponding author(s): Chris Sidey-Gibbons

Last updated by author(s): May 24, 2023

Reporting Summary

Nature Portfolio wishes to improve the reproducibility of the work that we publish. This form provides structure for consistency and transparency in reporting. For further information on Nature Portfolio policies, see our Editorial Policies and the Editorial Policy Checklist.

Statistics

For all statistical analyses, confirm that the following items are present in the figure legend, table legend, main text, or Methods section.

n/a	Confirmed	
☐	☒	The exact sample size (*n*) for each experimental group/condition, given as a discrete number and unit of measurement
☐	☒	A statement on whether measurements were taken from distinct samples or whether the same sample was measured repeatedly
☐	☒	The statistical test(s) used AND whether they are one- or two-sided<br>*Only common tests should be described solely by name; describe more complex techniques in the Methods section.*
☐	☒	A description of all covariates tested
☒	☐	A description of any assumptions or corrections, such as tests of normality and adjustment for multiple comparisons
☒	☐	A full description of the statistical parameters including central tendency (e.g. means) or other basic estimates (e.g. regression coefficient) AND variation (e.g. standard deviation) or associated estimates of uncertainty (e.g. confidence intervals)
☒	☐	For null hypothesis testing, the test statistic (e.g. *F*, *t*, *r*) with confidence intervals, effect sizes, degrees of freedom and *P* value noted<br>*Give P values as exact values whenever suitable.*
☒	☐	For Bayesian analysis, information on the choice of priors and Markov chain Monte Carlo settings
☒	☐	For hierarchical and complex designs, identification of the appropriate level for tests and full reporting of outcomes
☒	☐	Estimates of effect sizes (e.g. Cohen's *d*, Pearson's *r*), indicating how they were calculated

Our web collection on statistics for biologists contains articles on many of the points above.

Software and code

Policy information about availability of computer code

Data collection	Data were collected within the proprietary Epic electronic health record environment.
Data analysis	Analysis code can be accessed from the following repository: https://zenodo.org/record/7888547 (DOI : 10.5281/zenodo.7888547)

For manuscripts utilizing custom algorithms or software that are central to the research but not yet described in published literature, software must be made available to editors and reviewers. We strongly encourage code deposition in a community repository (e.g. GitHub). See the Nature Portfolio guidelines for submitting code & software for further information.

Data

Policy information about availability of data

All manuscripts must include a data availability statement. This statement should provide the following information, where applicable:

- Accession codes, unique identifiers, or web links for publicly available datasets
- A description of any restrictions on data availability
- For clinical datasets or third party data, please ensure that the statement adheres to our policy

Data Availability :
All data relating to the study are presented in the manuscript and supplementary materials. Patient data used to create, validate, and monitor our models are not publicly available due to ethical concerns. It may be possible to make data available as part of a future academic collaboration requiring separate institutional data collaboration agreements and additional IRB approval. Please contact Dr. C Gibbons to discuss. Source data for the figures are available as Supplementary Data 4.

Code Availability :
Analysis code can be accessed from the following repository: https://zenodo.org/record/7888547 (DOI : 10.5281/zenodo.7888547)

Human research participants

Policy information about studies involving human research participants and Sex and Gender in Research.

Reporting on sex and gender	Our reporting is consistent with the guidelines.
Population characteristics	See Above
Recruitment	Patients were not recruited into the study per se. We conducted an analysis of clinical data which was collected passively during patient care.
Ethics oversight	Ethics oversight was provided by the The University of Texas MD Anderson Cancer Center

Note that full information on the approval of the study protocol must also be provided in the manuscript.

Field-specific reporting

Please select the one below that is the best fit for your research. If you are not sure, read the appropriate sections before making your selection.

☐ Life sciences ☒ Behavioural & social sciences ☐ Ecological, evolutionary & environmental sciences

For a reference copy of the document with all sections, see nature.com/documents/nr-reporting-summary-flat.pdf

Behavioural & social sciences study design

All studies must disclose on these points even when the disclosure is negative.

Study description	We trained machine learning algorithms on routinely collected clinical data to predict 30-day ED admission.
Research sample	Patients attending clinical appointments at either the genitourinary or breast departments at a large cancer center
Sampling strategy	All patients were included in the analysis.
Data collection	Data were collected within the Epic EHR
Timing	Data were aggregated between Apirl 1990 and April 2022
Data exclusions	No exclusion were made.
Non-participation	N/A
Randomization	N/A

Reporting for specific materials, systems and methods

We require information from authors about some types of materials, experimental systems and methods used in many studies. Here, indicate whether each material, system or method listed is relevant to your study. If you are not sure if a list item applies to your research, read the appropriate section before selecting a response.

Materials & experimental systems

n/a	Involved in the study	
☒	☐	Antibodies
☒	☐	Eukaryotic cell lines
☒	☐	Palaeontology and archaeology
☒	☐	Animals and other organisms
☒	☐	Clinical data
☒	☐	Dual use research of concern

Methods

n/a	Involved in the study	
☒	☐	ChIP-seq
☒	☐	Flow cytometry
☒	☐	MRI-based neuroimaging
